# Ovicidal activity of spirotetramat and its effect on hatching, development and formation of *Frankliniella occidentalis* egg

**DOI:** 10.1038/s41598-021-00160-6

**Published:** 2021-10-21

**Authors:** Xiaomin Yang, Guiying Zhou, Lijuan Sun, Changying Zheng

**Affiliations:** College of Plant Health and Medicine, Qingdao Agricultural University, Qingdao, China

**Keywords:** Developmental biology, Ecology

## Abstract

*Frankliniella occidentalis* (Pergande) has become an important vegetable pest worldwide because of its economic damage to crop production. However, it is difficult to control due to its unique living habits. In this study, the eggs of *F. occidentalis* were used as the target to explore the ovicidal activity of spirotetramat on the thrips and its effect on hatching, development and formation. After the treatment of spirotetramat, the LC_50_ value descreased with increased egg age using egg dipping method, and showed the same trend as the leaf dipping method verified on living plants. Through ultra-depth-of-field microscopy, scanning electron microscopy and transmission electron microscopy, the egg shell and internal structures of *F. occidentalis* eggs were studied. Spirotetramat can destroy the egg shells of *F. occidentalis*, resulting in shrinkage of the egg surface, sunken pores, egg deformities, egg shell rupture and other phenomena. This allows spirotetramat to enter the egg and destroy the egg structure, making the egg internal structure flocculent, fuzzy and unevenly distributed, which affects embryonic development and causes the nymphs to die before hatching. Therefore, the prevention and control of *F. occidentalis* using spirotetramat before damage is caused to crops should have a better effect.

## Introduction

The western flower thrip, *Frankliniella occidentalis* (Pergande) (Thysanoptera: Thripidae), is among the world’s most important insect pests^1^. It has caused crop damage in Beijing, Shandong Province and other regions of China^2,3^, and is increasingly serious. *Frankliniella occidentalis* can directly feed on the young parts of crop plants and cause host yield reduction and death. At the same time, it can also spread Impatiens necrotic spot virus and Tomato spot wilt virus, causing serious indirect damage^4,5^. *Frankliniella occidentalis* are typical *r*-strategist insects. Once it occurs, the population increases exponentially.

Chemical pesticides are still the main method to control thrips in production, but because thrips have characteristics of small size, being well concealed, short generation cycle, rapid development, strong adaptability and easily generate resistance to pesticides, they are still difficult to control. At present, chemical control is mainly aimed at thrip nymphs and adults. Spirotetramat is a two-way systemic broad-spectrum insecticide, which can conduct up and down transmission in the plant with high efficiency and broad-spectrum, it is a fat synthesis inhibitor, which hinders the fat synthesis of insects and causes their poisoning and death. Previous studies showed that spirotetramat had low toxicity to nymphs and adults of *F. occidentalis*, and almost no direct lethal effect. However, spirotetramat can significantly restrain *F. occidentalis* population development, and population parameters such as parental life span, offspring nymph number, adult number, eclosion rate and female-to-male ratio decrease significantly^6^. The egg laying of *F. occidentalis* is not easily observed in plant tissues, and so is difficult to study, resulting in few studies on eggs as the target of insecticides. Our previous studies showed that spirotetramat could affect the growth, development and reproduction of *F. occidentalis*^7^. The development duration, survival rate and related population dynamics of *F. occidentalis* were adversely affected by sublethal dose stress^7^. Kateřina Kovaříková also found that spirotetramat had good ovicidal activity against *Aleyrodes proletella*^8^.

Studies have reported that insecticides can cause changes in egg embryo development and egg shell morphology^9,10^. Insect egg shell is mainly composed of egg shell layer and yolk membrane, and different insects have differing egg shell structures. Egg shells and biofilms are barriers to prevent entry of toxic compounds, and in order to be effective chemical pesticides must break through these barriers to enter the eggs. Two insecticides, methomyl and hydrochloride, destroyed the inner egg shell of *Neoleucinodes elegantalis*, inhibited embryonic development and eventually caused the larvae to die before hatching^11^. Water-soluble *Moringa oleifera* lectin was used to treat the eggs of *Aedes aegypti*; after 72 h, the egg shell surface was severely deformed and degraded, and the egg shell broke and the egg did not hatch^12^. Our earlier experiments showed that after spirotetramat treatment, the eggs of *F. occidentalis*, the eggs became sticky and soft, and white cotton-like agglomerates appeared around the eggs, which did not develop normally. We speculate that spirotetramat may have damaged *F. occidentalis* egg shell structure or egg shell composition. However, if spirotetramat was applied after eggs had developed for a period of time, there was no obvious difference in egg surface morphology. It has been reported that the *Anopheles* mosquito eggs are initially soft and white, but as the embryo develops, the sclera in the shell hardens and blackens^13^. After a period of embryonic development, the eggshell structure may change, which may result in drug resistance.

In this study, we explored *F. occidentalis* eggs were used as insecticide explore the indoor toxicity, embryonic development and egg shell morphology changes caused by spirotetramat on freshly laid *F. occidentalis* eggs and following some development. The mode of action of spirotetramat on *F. occidentalis* eggs provides a theoretical basis for the green prevention and control of *F. occidentalis*.

## Materials and methods

### Insects

*Frankliniella occidentalis* individuals were initially collected from *Trifolium repens* L. (Fabales: Fabaceae) on the campus of Qingdao Agricultural University (QAU), Qingdao, China, in 2007, and then continuously reared in the insect culture room of QAU on kidney bean, *Phaseolus vulgaris* L. (Fabales: Fabaceae). The rearing conditions were set at 25 ± 1 °C, 60% ± 10% relative humidity and a photoperiod of 16:8 h (light:dark).

### Toxicity test and validation

#### Collection of *F. occidentalis* eggs in vitro

The patented method for collecting *F. occidentalis* eggs (Patent no. ZL 2015 1 0519793.2, CN105210997A)^14^, was used to collect eggs for the toxicity test and the following study. The bottom of a two-compartment plastic Petri dish (12 cm in diameter) was cut out and then covered with stretched parafilm (original size 3–4 cm^2^). *Frankliniella occidentalis* adults were transferred onto the parafilm, and then the upper side was sealed with plastic wrap. Insect pins (No. 0) were used to punch holes in the plastic wrap for ventilation. The cover of a glass Petri dish of the same size was placed upside down and filled with distilled water to half of its height. The parafilm side of the plastic Petri dish was placed into the glass Petri dish cover with the parafilm side down on the water. Thrips can use their ovipositor to penetrate the parafilm and lay eggs in the water. After 24 h, adult feeding containers were removed and eggs were collected from the water; at this point an egg is a “0-h-old eggs”, an egg after 12 h is a “12-h-old eggs” and an egg after 24 h is a “24-h-old eggs”.

#### *Frankliniella occidentalis* eggs collected from living plants

The egg collection device of *F. occidentalis* at 0 h, 12 h and 24 h was made from a disposable transparent plastic cup. The bottom side of the cup was removed and then covered with 200-mesh gauze and sealed with a glue gun. A disposable wooden chopstick was fixed with a glue gun near the bottom of the cup outside the cup wall, to make a closed chamber for collection of *F. occidentalis* eggs. The egg collecting device was fitted to the leaves of kidney bean plants, and the top was sealed with preservative film and secured with a rubber band. Fifteen pairs of *F. occidentalis* adults that experienced eclosion on the same day were placed into one collection device; The adults lay their eggs in the leaf tissue and are removed after 24 h, at which point the leaf is a leaf with *F. occidentalis* eggs. At this point an egg is a “0-h-old eggs”, an egg allowed to develop for 12 h on a kidney bean plant is a “12-h-old eggs” and an egg allowed to develop for 24 h is a “24-h-old eggs”.

#### Toxicity test of eggs in vitro

The toxicity of spirotetramat to *F. occidentalis* at 0-h-, 12-h- and 24-h-old eggs was determined by the method of egg dipping method. According to the preliminary test results, the test agent was first dissolved in acetone to form a 100 mg/L stock solution, then diluted with distilled water to five gradient concentrations, mixed well and set aside. The collected eggs were transferred to glass Petri dishes (diameter 35 mm) containing different concentrations of agents with a 2.5-μL pipette under a Leica M80 binocular microscope (Germany). Twenty *F. occidentalis* eggs were evenly distributed in each Petri dish, to avoid aggregation and adhesion. The Petri dish was cultured indoors under the same conditions as described in Sect. 2.1. There were four replicates for each concentration, and the control group was treated with clear water and acetone. The hatching of eggs was observed and record Leica M80 binocular microscope. Observation continued for 144 h, the condition of eggs was observed per 24 h, and the number of eggs that did not hatch were recorded. The eggs that did not hatch after 144 h treated by spirotetramat were considered as dead.

#### Toxicity test of eggs on living plants

In the field, *F. occidentalis* eggs are laid in plant tissues using a sawing ovipositor, so it is hard to collect many eggs for testing. In this study, the toxicity of spirotetramat on *F. occidentalis* eggs on live plants was determined (by leaf dipping method) to verify the method of collecting *F. occidentalis* eggs in vitro. The spirotetramat was applied using the same five gradient concentrations as in 2.2.3. The leaves of kidney bean plants with *F. occidentalis* eggs were immersed in full contact with the working solution for 30 s, removed and allowed to dry naturally; 24 h later remove the leaves from the plants and placed in a Petri dish containing agar (1.5% agar, with a layer of filter paper added to moisturize after the agar solidified) and sealed with plastic wrap, which was punctured with a dissecting needle to provide ventilation. There were four replications for each concentration, and the control group was treated with clear water and acetone. The number of newly emerged nymphs on leaves with eggs in 144 h were recorded. Assuming that the control group all hatched, the relative mortality of each concentration was calculated.

#### Data processing

Data of toxicity test were analyzed according to Probit analysis^15^ by using the computer program Probit-MSChart^16^.

### Egg morphology and structure observations

#### Test treatment setup

Indoor toxicity testing showed that susceptibility of *F. occidentalis* eggs to spirotetramat differed at different developmental stages: 24-h-old eggs were less sensitive than 0-h-old eggs. Therefore, in this test set, the treatment concentration of spirotetramat was the LC_50_ value of 12-h-old eggs, that is 14.418 mg/L. The purpose was to select a higher concentration of spirotetramat for 0-h-old eggs, for the lower concentration of spirotetramat for 24-h-old eggs, and explore in more detail of the effect of spirotetramat on external morphology and shell structure of *F. occidentalis* eggs.

#### Egg external shape observations

Eggs of *F. occidentalis *in vitro and the eggs on living plants were collected according to 2.2.1 and 2.2.2. They were treated with spirotetramat using egg-dipping and leaf-dipping methods mentioned in 2.2.3 and 2.2.4, repsectively. The spirotetramat concentration was as used in 2.1.3 of 12 h LC_50_ (14.418 mg/L); after 24 h of spirotetramat treatment, the detached eggs were directly placed under an ultra-depth-of-field microscope (VHX-2000, Keyence, Shanghai, China) to observe and take pictures. For eggs on live plants, under the Leica M80 binocular microscope, a No. 0 insect needle was used to cut the surface of kidney bean leaves and the leaf surface was gently lifted to expose the eggs, and then plant tissue around the egg was meticulously peeled away. The eggs were lightly touched with the insect needle shaft to make them adhere to the shaft, and then moved to a glass Petri dish (diameter 35 mm) filled with distilled water, observed and imaged under an ultra-depth-of-field microscope.

#### Impact of egg hatchability

One-hundred *F. occidentalis* eggs in vitro and on live plants were treated with spirotetramat as in 2.3.2, then allowed to grow and develop in the working solution. The condition of eggs was observed daily and the number of eggs that did not hatch when the shell was broken, the number of eggs that did not hatch when the shell was not broken (eggs not hatched within 144 h) and the number of eggs that hatched normally were recorded. The control group was treated with clear water and acetone.

#### Scanning electron microscope (SEM) observations

According to the method in 2.2.1, sufficient 0 and 24 h *F. occidentalis* eggs were collected under a dissecting microscope, and transferred with a 2.5-μL pipette to 14.418 mg/L spirotetramat solution. After 24 h, multiple eggs were fixed in 2.5% glutaraldehyde for more than 4 h under a dissecting microscope, the fixative was poured out and the samples were rinsed three times with 0.1 M, pH 7.0 phosphate buffer, for 15 min each time. The sample was fixed with 1% osmium acid solution for 1–2 h, the osmium acid waste liquid was carefully removed and the sample was rinsed three times with 0.1 M, pH 7.0 phosphate buffer, for 15 min each time. The samples were dehydrated with ethanol solution at each concentration of 30%, 50%, 70%, 80%, 90% and 95% for 15 min. Then, samples were treated twice with 100% ethanol for 20 min each time, with a mixture of ethanol and isoamyl acetate (V/V = 1/1) for 30 min and then with pure isoamyl acetate for 1 h or left overnight. The resulting samples are critical point drying at the coating film. The processed sample was observed using an SEM.

#### Transmission electron microscope (TEM) observations

Collection and handling of eggs, fixation, rinsing and dehydration of samples were same as described in Sect. 2.3.4. Then with pure acetone for 20 min. Samples were treated with a mixture of embedding agent and acetone (V/V = 1/1) for 1 h, a mixture of embedding agent and acetone (V/V = 3/1) for 3 h and finally pure embedding agent overnight, after which the infiltrated samples were heated overnight at 70 °C to obtain embedded samples. The sample was sliced in a ultra-thin microtome (LEICA EM UC7) to obtain 70–90 nm slices, the sections were stained with lead citrate solution and uranyl acetate 50% ethanol saturated solution for 5–10 min and then dried for observation using a TEM.

#### Effect on embryonic development

Eggs of *F. occidentalis* were collected according to 2.2.1. Spirotetramat was applied at the LC_50_ (14.418 mg/L) using the egg dipping method for 12 h to individual collected eggs, and the entire developmental process photographed for the same egg. Egg morphological characteristics were observed every 12 h, and photomicrographs were taken under the VHX-2000 ultra-depth-of-field microscope. The control group was treated with clear water and acetone.

### Ethics approval

Approvals.

### Consent to participate

Consent.

### Consent for publication

Consent.

## Results and analysis

### Toxicity of spirotetramat to *F. occidentalis* eggs

The indoor toxicity of spirotetramat to 0-h-, 12-h-, and 24-h-old eggs of *F. occidentalis* using egg dipping method and leaf dipping method was shown in Table 1 after egg hatching was observed for 144 h. The results suggested that the LC_50_ value gradually decreased as the egg age increased. The two methods have the same trend. And in the leaf dipping method, according to the confidence limits analysis, 0-h-old eggs are significantly more sensitive to spirotretramat than 24-h-old eggs.

**Table 1:** Toxicity of spirotetramat to *F. occidentalis* eggs*.*

Method	Egg age (h)	Toxicity regression	LC_50_ (mg/L)	95% Confidence limits (mg/L)
Egg dipping method	0	y = 2.851 + 2.350x	8.549	2.980–22.740
	12	y = 1.141 + 2.550x	14.418	8.092–30.459
	24	y = 1.606 + 1.915x	32.471	13.549–71.362
Leaf dipping method	0	y = 2.504 + 1.408x	12.712	10.556–19.556
	12	y = 2.871 + 1.385x	15.888	15.118–41.368
	24	y = 2.882 + 0.929x	51.856	26.219–105.634

### Egg external shape observations

**Figure 1:**
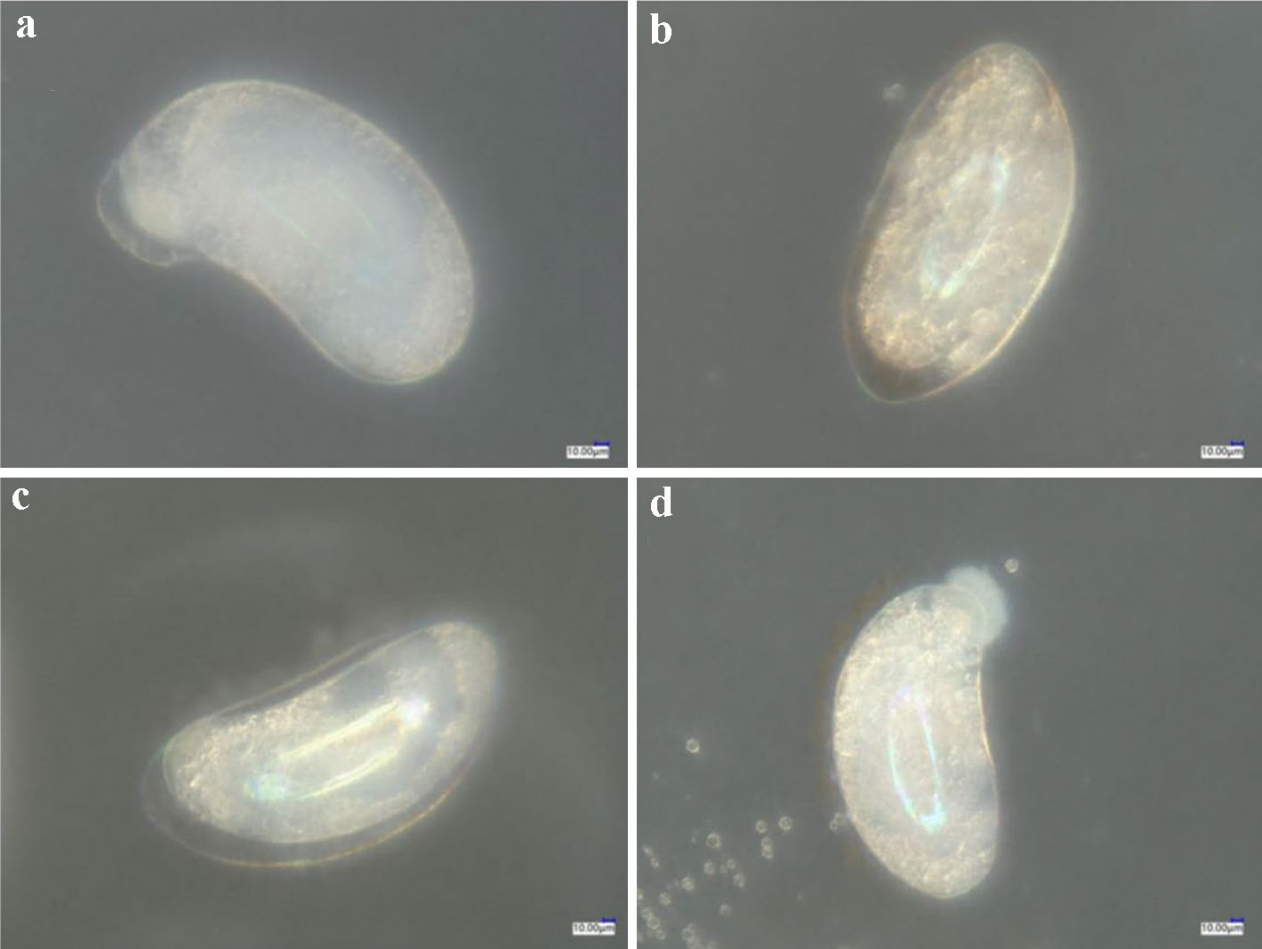
The effect of spirotetramat on external morphology of isolated 0-h-old eggs of *F. occidentalis*. (**a**) 0-h-old isolated eggs of normal developing thrips; (**b**) yellowish-brown, developmentally deformed eggs after spirotetramat treatment; (**c**) eggs with shrunken oocytes after spirotetramat treatment; (**d**) eggs with apical rupture of the eggshell after spirotetramat treatment.

External morphology of normally developed isolated 0-h-old eggs of *F. occidentalis* in the control treatment (Fig. 1a) were compared with those treated with spirotetramat (Fig. 1b, c). After spirotetramat treatment, some eggs appeared darker, yellowish-brown, and with embryonic development abnormalities (Fig. 1b); some of the egg embryo cells treated with spirotetramat appeared atrophied, and there were obvious gaps between the outer and the inner egg embryo cells, compared with the control treatment (Fig. 1c). Some eggs ruptured at the top after the egg shell was treated with spirotetramat, and the internal egg embryo cells flowed out, thus failing to form a complete embryo (Fig. 1d); the full egg embryo cells formed a control treatment.

**Figure 2:**
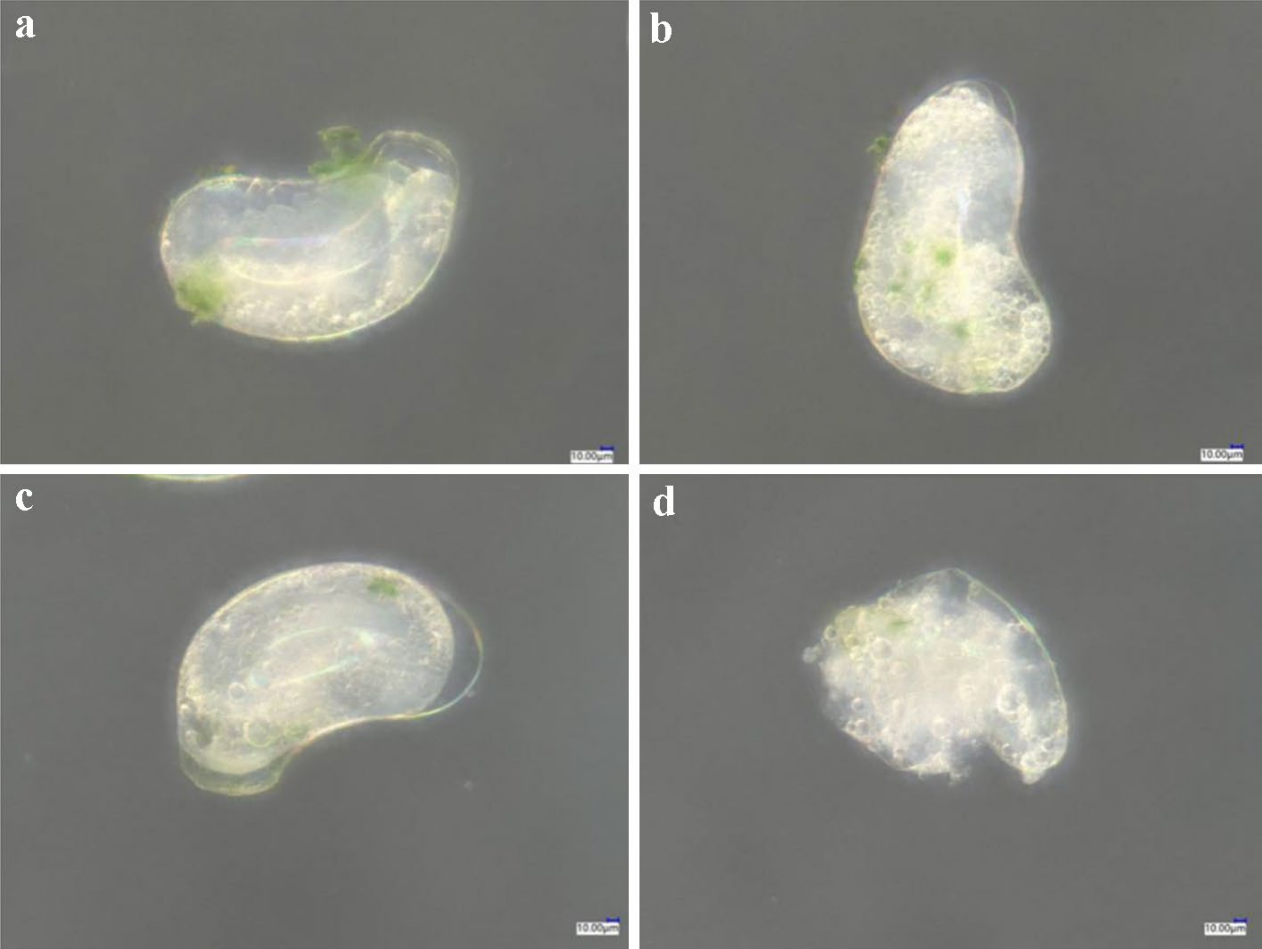
The effect of spirotetramat on external morphology of living 0-h-old eggs of *F. occidentalis*. (**a**) External morphology of live 0-h-old eggs of normal developing thrips; (**b**) developmentally deformed eggs after spirotetramat treatment; (**c**) eggs with shrunken oocytes after spirotetramat treatment; (**d**) eggs with apical rupture of the eggshell after spirotetramat treatment.

The effect of spirotetramat on external morphology of live *F. occidentalis* 0-h-old eggs (Fig. 2b, c) was compared with normal development in the control treatment (Fig. 2a). Similar to the effect on external morphology of isolated 0-h-old eggs of *F. occidentalis*, the eggs treated with spirotetramat also showed abnormal embryonic development (Fig. 2b), egg embryo cell atrophy (Fig. 2c) and the phenomenon of rupture of the egg shell and outflow of embryo cells.

**Figure 3:**
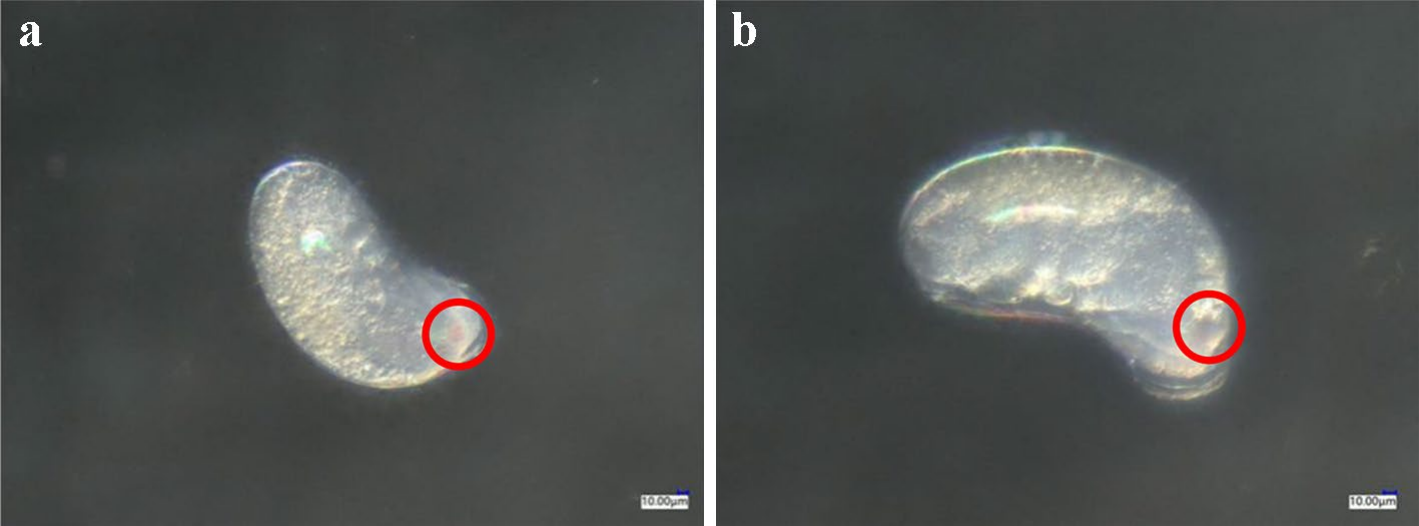
The effect of spirotetramat on external morphology of isolated 24-h-old eggs of *F. occidentalis*. (**a**) Normally developing 24-h-isolated eggs in the control group; (**b**) 24-h-old eggs after spirotetramat treatment.

**Figure 4:**
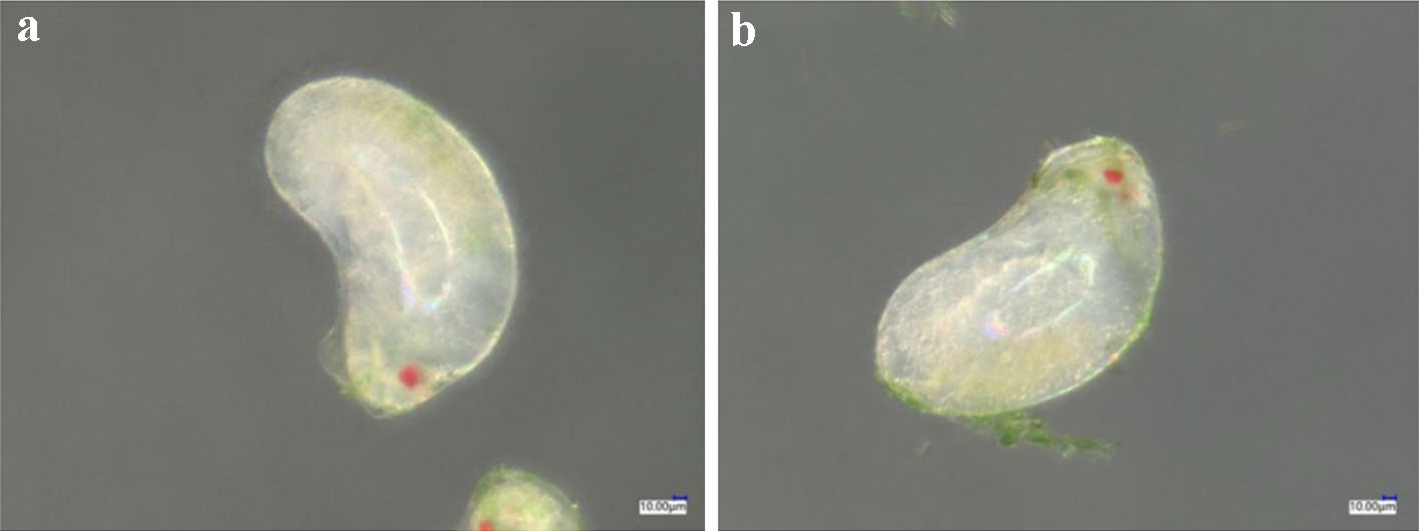
The effect of spirotetramat on external morphology living of 24-h-old eggs of *F. occidentalis*. (**a**) Normally developing 24-h-live eggs in the control group; (**b**) live 24-h-old eggs after spirotetramat treatment.

Compared with the control (Fig. 3a), the isolated 24-h-old eggs treated with spirotetramat (Fig. 3b) did not show obvious external morphological differences. After spirotetramat treatment, the eggs were still white and plump and with no embryonic deformities, egg cell atrophy or egg shell rupture, and could still develop normally. There were clear red eye spots on the head end, and embryo movement was clearly seen under the super-depth microscope (Fig. 3b). Similarly, the live 24-h-old eggs in the control treatment (Fig. 4a) and those treated with spirotetramat (Fig. 4b) showed no obvious external morphological differences, and the eggs developed normally.

### Effect of egg hatching

**Figure 5:**
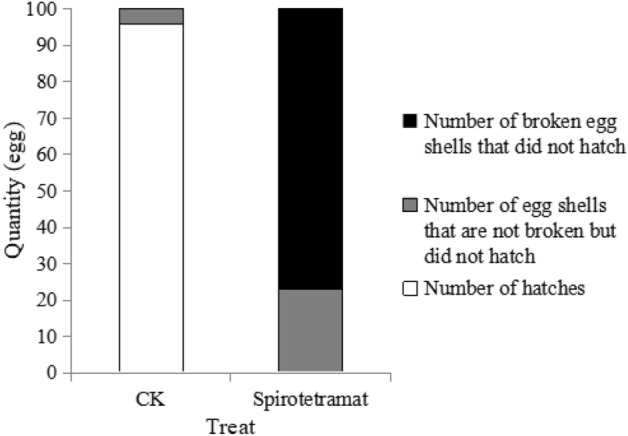
Effect of spirotetramat on hatching rate of *F. occidentalis* 0-h-old eggs.

The 0-h-old eggs of *F. occidentalis* treated with spirotetramat did not hatch normally, and the mortality rate was 100% (Fig. 5). Among them, 77 eggs eventually showed rupture of the egg shell, the internal egg embryo cells flowed out and they did not hatch; 23 eggs showed no changes in external morphology, but did not hatch after continuous observation for 144 h, and showed no developmental phenomena such as embryo movement under a super-depth microscope, which was regarded as egg death. In the control treatment, 96 eggs hatched normally, and only six eggs did not rupture but did not hatch normally and were considered dead.

**Figure 6:**
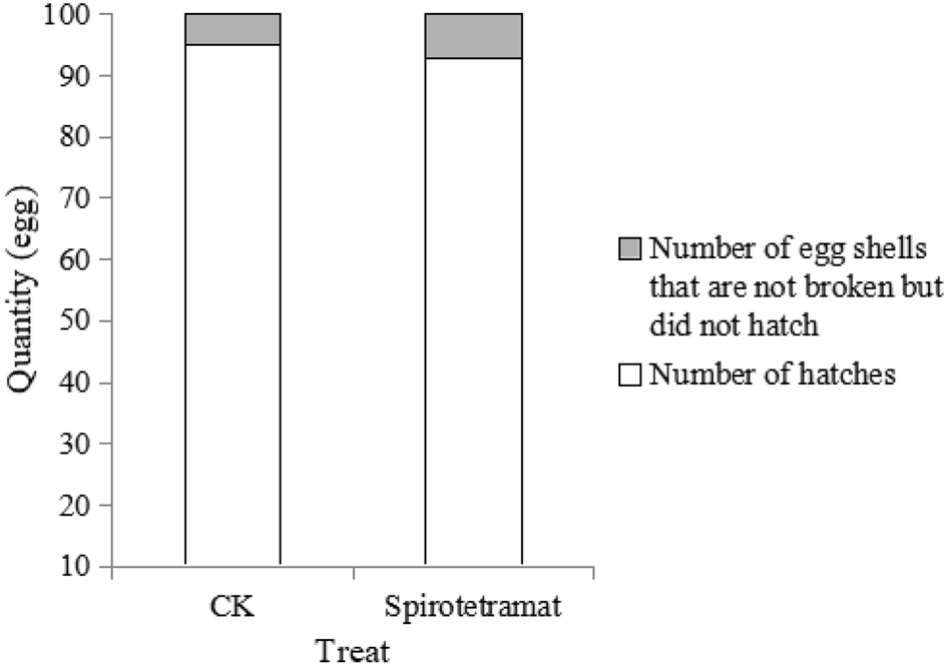
The effect of spirotetramat on hatching rate of *F. occidentalis* 24-h-old eggs.

There was no significant difference between the 24-h-old eggs of *F. occidentalis* treated with spirotetramat and the control treatment. After spirotetramat treatment, 93 eggs hatched normally, and the shells of seven eggs were not ruptured (Fig. 6). Any eggs not hatched after 144 h of continuous observation were considered dead. In the control treatment, 95 eggs hatched normally and five eggs did not rupture but did not hatch normally, and so were considered dead.

### SEM observations

**Figure 7:**
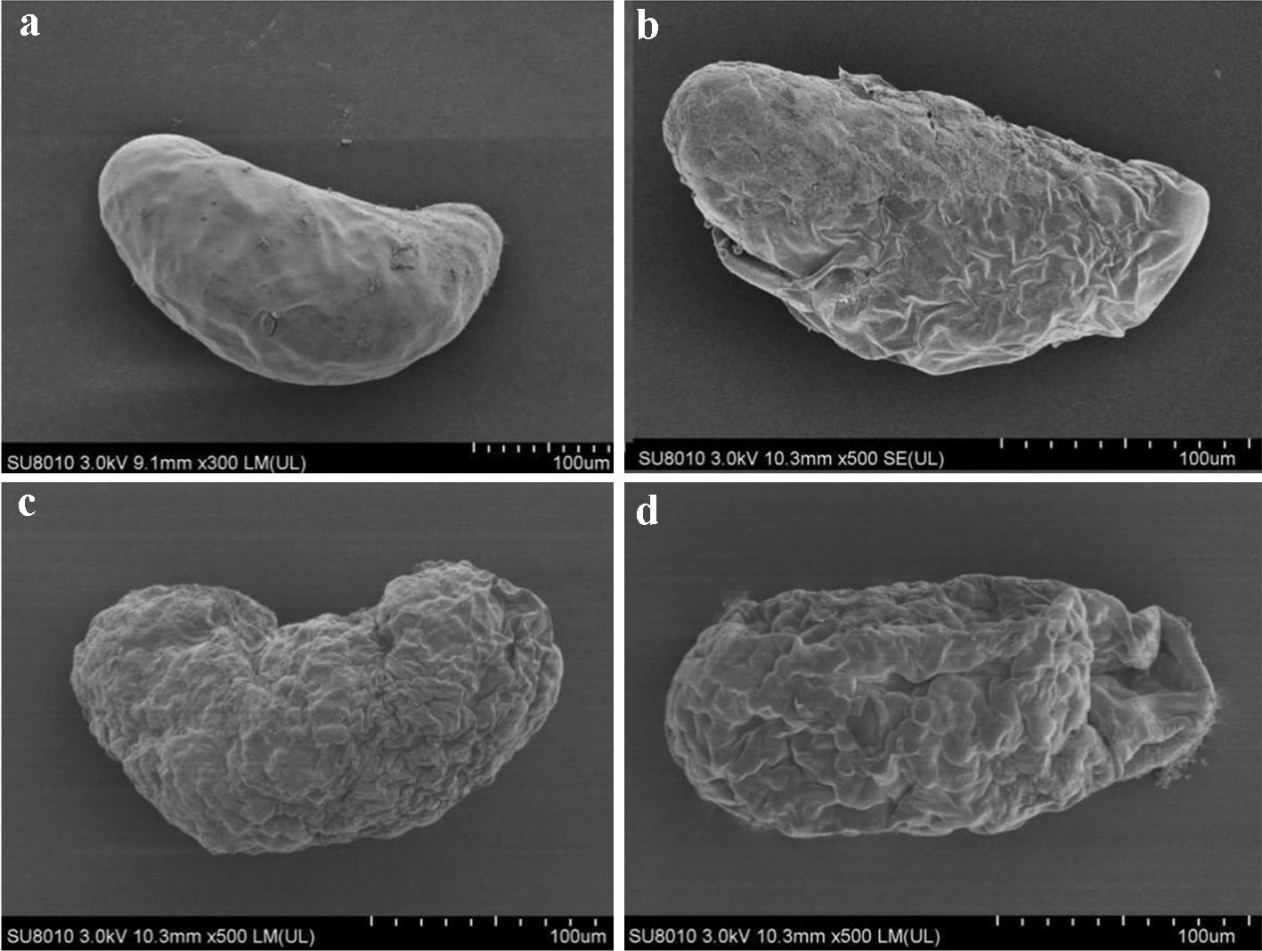
The effect of spirotetramat on the surface of egg shells of *F. occidentalis* 0-h-old eggs. (**a**) 0-h-old eggs in the control treatment; (**b**) eggs shells were shed 24 h after treatment with spirotetramat; (**c**) the surface of the egg shell was uneven and severely wrinkled; (**d**) the pores of some eggs were sunken down and shrunken after treatment with spirotetramat.

The *F. occidentalis* eggs in the control treatment were kidney-shaped, with regular egg morphology, smooth surfaces and no folds or protrusions (Fig. 7a). At 24 h after spirotetramat treatment, part of the egg shells treated with spirotetramat had fallen off the chorion, and the embryonic material was exposed (Fig. 7b). The surface of the egg shell was uneven and severely wrinkled (Fig. 7c). The pores of some eggs treated with spirotetramat were sunken down and shrunken (Fig. 7d). Spirotetramat treatment of 0-h-old eggs affect clearly egg shells, resulting in shrinkage of egg shells, ovarian depression and egg malformations, and destroyed the egg shell structure. Thus, normal embryonic development was affected, and disrupted normal hatching.

**Figure 8:**
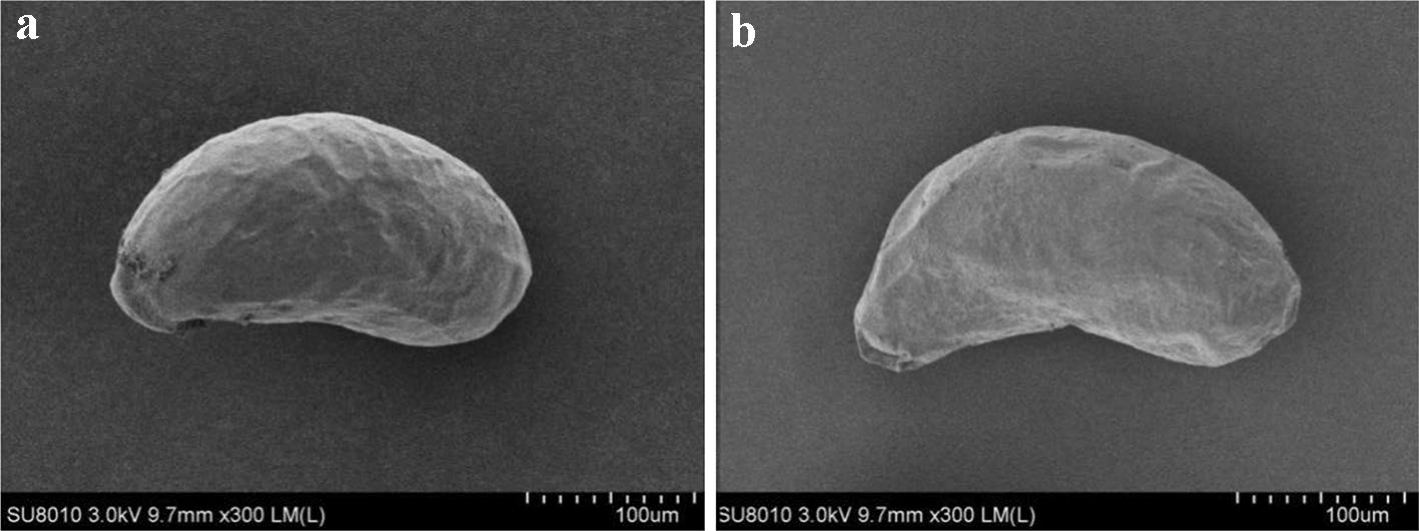
The effect of spirotetramat on the egg shell surface of *F. occidentalis* 24-h-old eggs. (**a**) 24-h-old eggs in the control treatment; (**b**) 24-h-old eggs in the spirotetramat treatment.

The shells of eggs treated with spirotetramat (Fig. 8b) showed no significant difference compared with controls (Fig. 8a). The eggs of the two groups of *F. occidentalis* were regular in shape, with smooth surfaces and without folds or protrusions. Thus, development of 24-h-old eggs showed some resistance to spirotetramat. Spirotetramat did not destroy the egg shell surface structure of 24-h-old eggs, indicating a high resistance to spirotetramat.

### TEM observations

**Figure 9:**
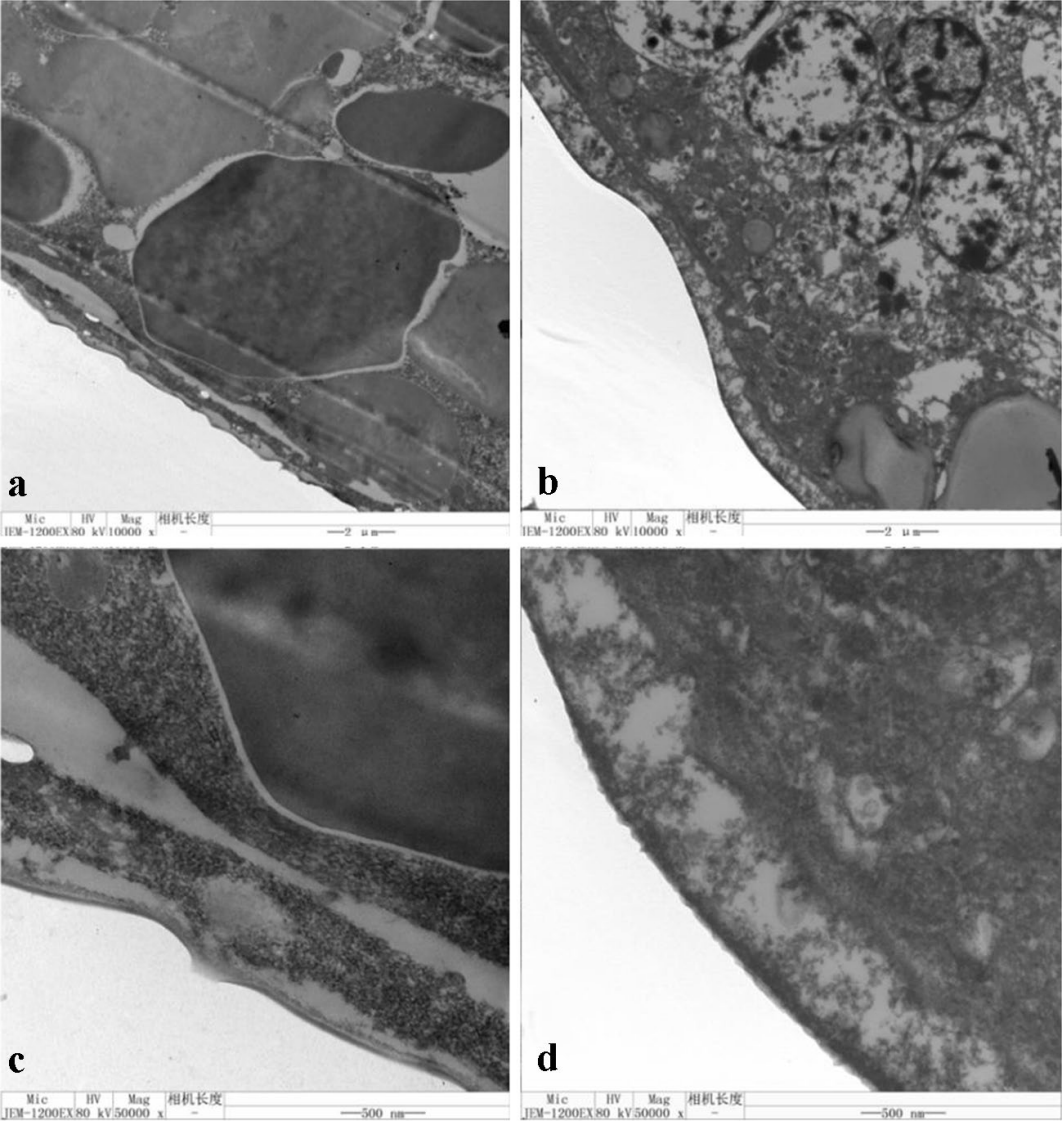
The effect of spirotetramat on the structure of *F. occidentalis* 0-h-old eggs. (**a**) and (**b**) 0-h-old eggs in the control treatment; (**c**) and (**d**) 0-h-old eggs in the spirotetramat treatment.

The TEM observations showed that the egg structure of the control treatment was complete, the protoplasm and yolk were clearly observed inside the egg and the yolk was packed in the void of the protoplasm network (Fig. 9a). The egg shell structure was clear, and the outer and inner egg shell were clearly observed, as was the yolk membrane and the dense layer structure (Fig. 9c). Eggs treated with spirotetramat were flocculent, and no clear internal material was observed. The protoplasm and yolk structure were blurred, and flocculation in the protoplasm appeared to agglomerate and form blocks (Fig. 9b). The egg shell structure was unclear, and no clear outer egg shell, inner egg shell, yolk membrane and lamellar structures were observed. The egg shell was also filled with many flocs (Fig. 9d).

### Effect on embryonic development

**Figure 10:**
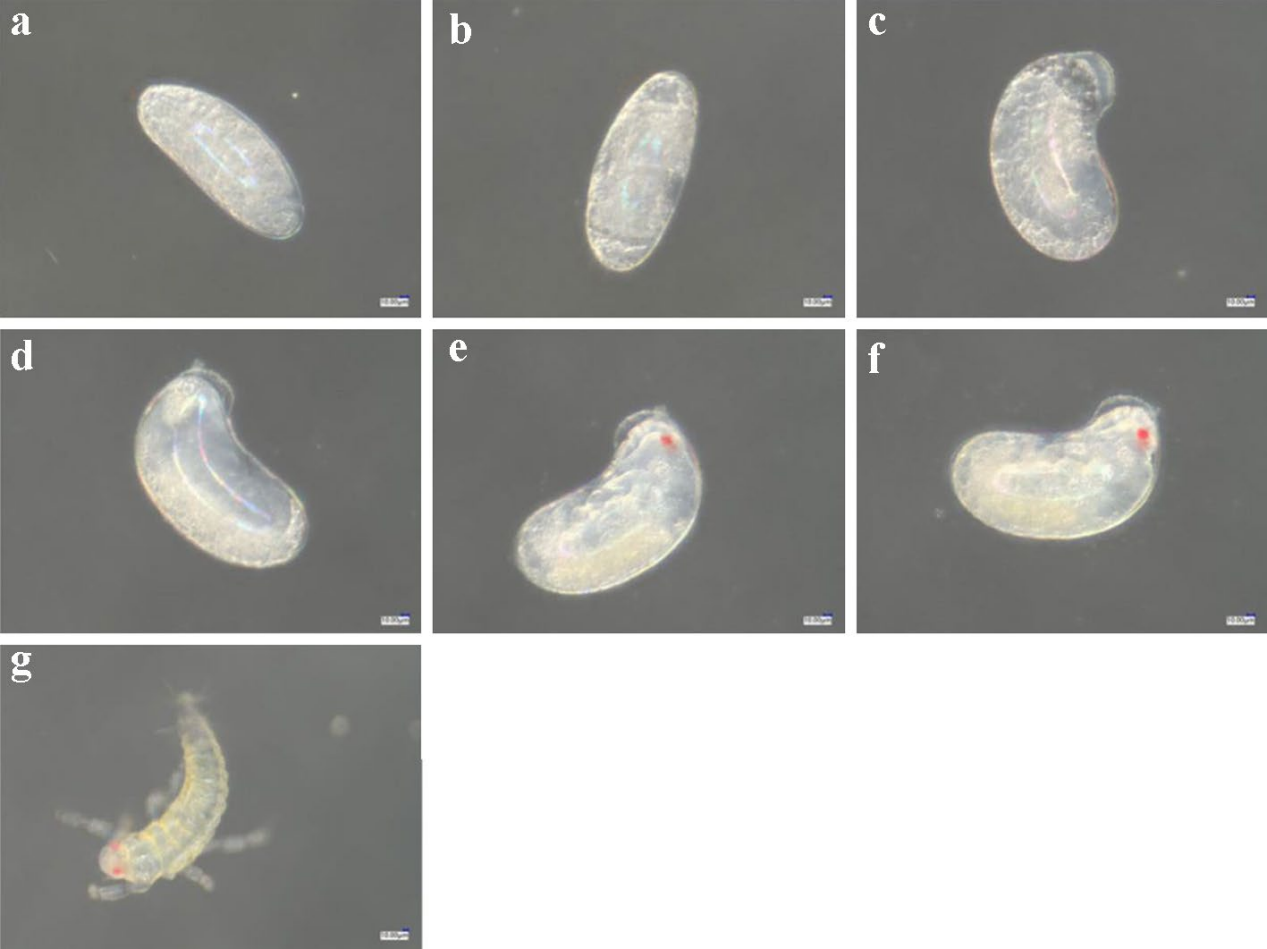
The embryonic development process of control 0-h-old eggs of *F. occidentalis*. (**a**) Control initial eggs; (**b**) eggs after 12 h of development; (**c**) eggs after 24 h of development; (**d**) eggs after 36 h of development; (**e**) eggs after 48 h of development; (**f**) eggs after 60 h of development; (**g**) eggs hatching as larvae after 72 h of development.

The initial eggs of the control group were kidney-shaped, white and full of vitellin (Fig. 10a). After 12 h of development, the eggs were larger and of oval shape (Fig. 10b). After 24 h of development, the egg had increased in volume, a partially transparent region appeared in the embryo and the embryo had transparent top follicles (Fig. 10c). After 36 h of development, some yolk granules disappeared and eggs became smooth and translucent (Fig. 10d). After 48 h of development, the insect outline was visible within the egg, a pair of antennae were visible on the head and a red eye point was clearly observed on the head during the blastokinesis phenomenon (Fig. 10e). After 60 h of development, embryo color deepened, the eye point was clearer and the head, femur, tibia and tarsus were clear (Fig. 10f). After 72 h of development, the egg shell began to break at the head, the tail constantly jittered, internal fluid flowed and the larva hatched from the top of the egg (Fig. 10g).

**Figure 11:**
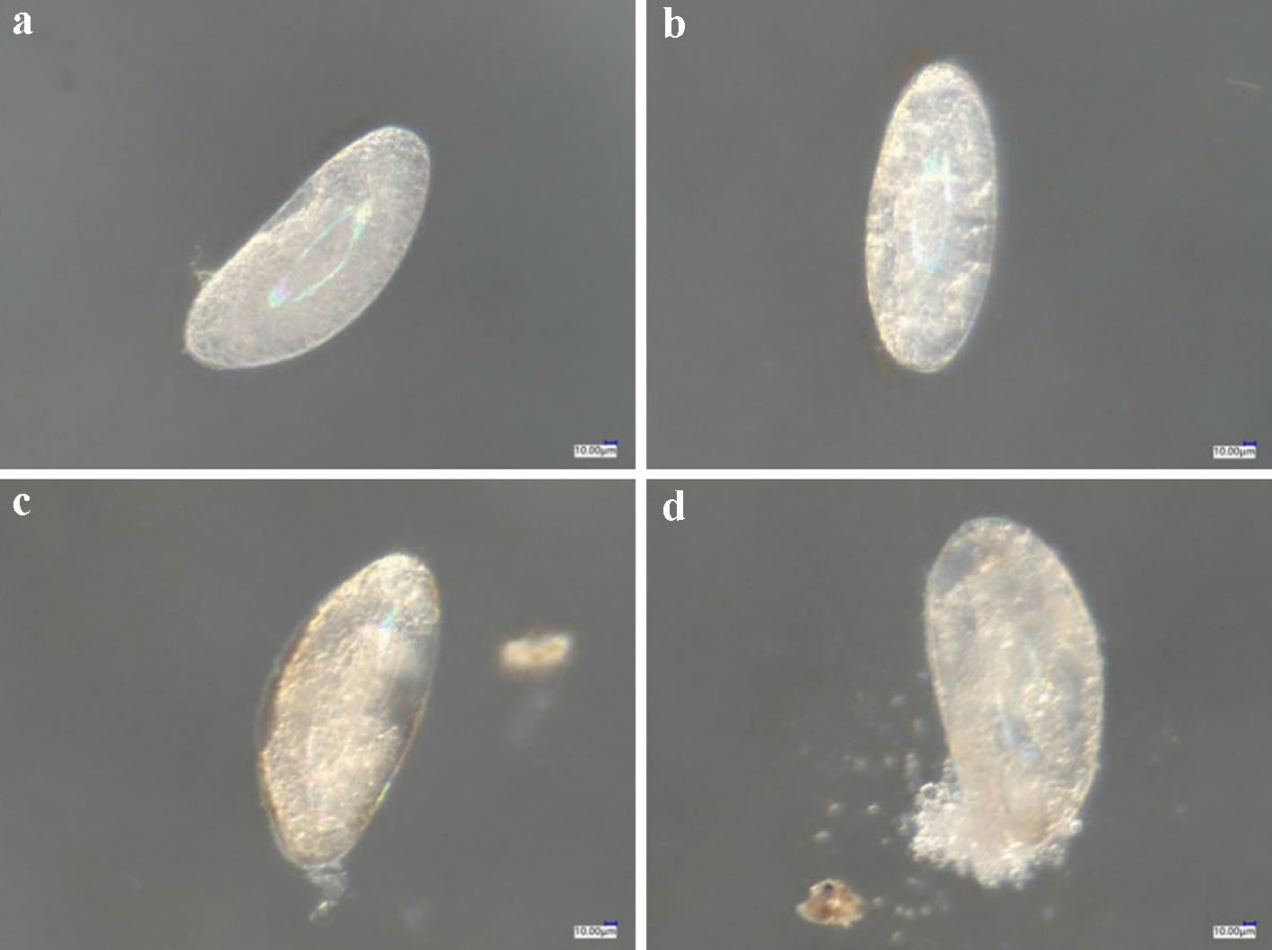
Effects of spirotetramat on development of 0-h-old eggs of *F. occidentalis.* (**a**) *Frankliniella occidentalis* initial eggs; (**b**) eggs developing 12 h after spirotetramat treatment; (**c**) eggs developing 24 h after spirotetramat treatment; (**d**) eggs developing 36 h after spirotetramat treatment.

Eggs of *F. occidentalis* were initially white, kidney-shaped and full of vitellin (Fig. 11a). Following treatment with spirotetramat, after 12 h of development, the eggs became large and oval, and the embryo was a pale brown color (Fig. 11b). After 24 h of development, color of the egg deepened to dark brown. There was a gap between the egg and the egg shell, and a small amount of spillage appeared at the end of the egg (Fig. 11c). After 36 h of development, the egg shell ruptured, material flowed out of the egg and embryo development did not proceed (Fig. 11d).

**Figure 12:**
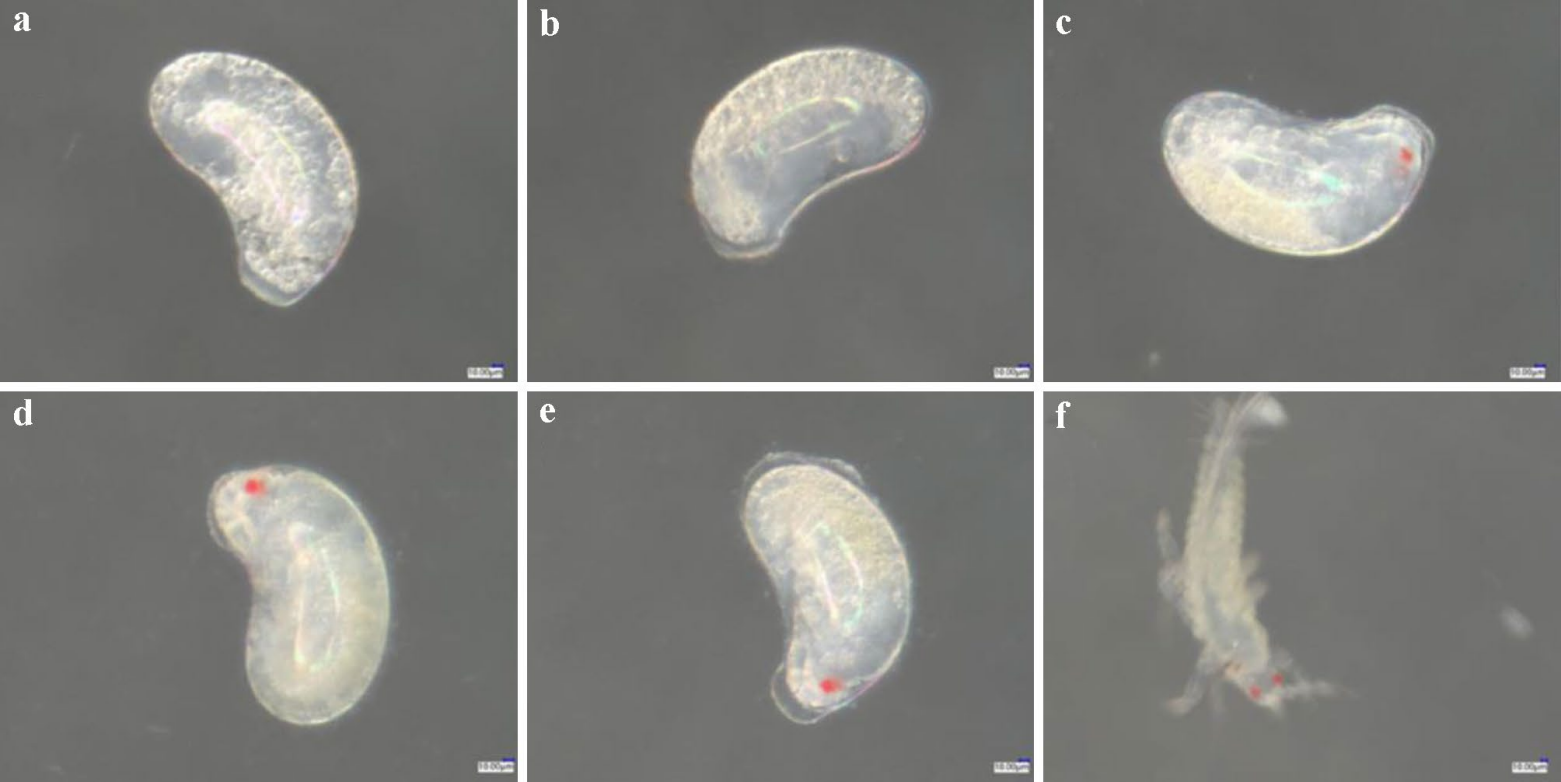
24-h-old eggs embryo development of *F. occidentalis* in control treatment. (**a**) Control 24-h-old eggs; (**b**) eggs after 12 h of development; (**c**) eggs after 24 h of development; (**d**) eggs after 36 h of development; (**e**) eggs after 48 h of development; (**f**) eggs hatching as larvae after 60 h of development.

In the control treatment, the egg volume increased at 24 h, the embryo had a partially transparent area and there was a transparent follicle on the top of the embryo (Fig. 12a). After 12 h of development, some of the yolk particles disappeared and the egg body was smooth and translucent (Fig. 12b). After 24 h of development, the body outline, a pair of antennae and red eye spots were visible, and there was obvious embryo movement (Fig. 12c). At 36 h of development, the head, leg segments, tibia and tarsus were apparent (Fig. 12d). After 48 h of development, the embryo moved violently, internal body fluid flowed and the larva was ready to hatch (Fig. 12e). After 60 h of development, the larva emerged from its shell (Fig. 12f).

**Figure 13:**
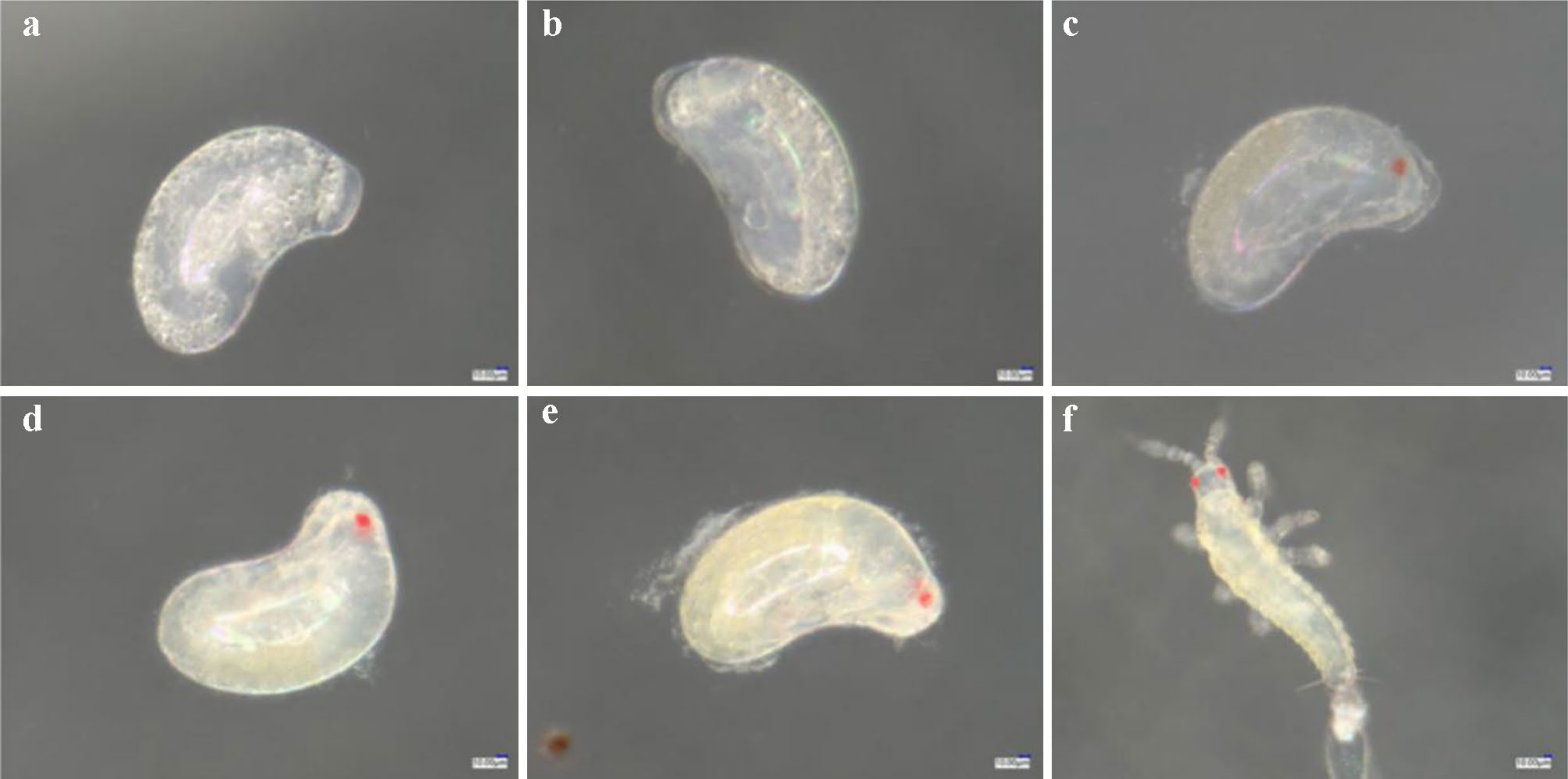
The effect of spirotetramat on embryonic development of *F. occidentalis* 24-h-old eggs. (**a**) *Frankliniella occidentalis* 24-h-old eggs; (**b**) eggs developing 12 h after spirotetramat treatment; (**c**) eggs developing 24 h after spirotetramat treatment; (**d**) eggs developing 36 h after spirotetramat treatment; (**e**) eggs developing 48 h after spirotetramat treatment; (**f**) eggs hatching as larvae after 60 h of development.

The 24 h old eggs of *F. occidentalis* showed enlarged volume, and there were transparent follicles on the top of the embryo (Fig. 13a). After 24 h eggs were treated with spirotetramat, they developed for 12–36 h, and the developmental status was the same as that of the control. The embryos developed normally, and there was no egg body discoloration or egg shell rupture (Fig. 13b–d). After 48 h of development, hairy scales appeared on the surface of the egg shell, and the egg body turned yellowish-brown in color, but the egg shell was not broken and no internal material overflow was seen (Fig. 13e). After 60 h of development, larvae hatched normally (Fig. 13f).

## Discussion

Chemical control of *F. occidentalis* mainly targets nymphs or adults. Studies have shown that some commonly used bio-sources, pyrethroids, neonicotinoids and insect growth regulators have insecticidal activity against nymphs and adults of *F. occidentalis* in the field^17,18^. However, nymphs and adults often lurk in the overlapping areas of buds, stamens and petals, and are well concealed; adults crawl quickly and it is generally difficult for general insecticides to directly contact them^19^. In this experiment, we used *F. occidentalis* eggs as the research target. The results showed that spirotetramat had high toxicity to eggs. With increased egg age, the LC_50_ value to spirotetramat decreased gradually. *Frankliniella occidentalis* are produced in plant tissues. In order to apply the treatment of isolated eggs to actual production conditions, this experiment was verified by a living plant method. The results showed the same trends for the live plant and egg dipping methods. Spirotetramat showed high toxicity, and the LC_50_ value gradually decreased with increased egg age. However, the in vivo plant method resulted in a higher LC_50_ than the egg dipping method, possibly due to material transportation and metabolism of the plant itself.

Both methods were used to explore the effect of spirotetramat on the external morphology of *F. occidentalis* eggs. The first eggs of *F. occidentalis* were treated with two methods, and the egg shells broke, the contents leaked and the eggs were deformed and unable to continue developing (Figs. 1, 2). The eggs that were treated with spirotetramat after 24 h of embryonic development showed no significant difference in external morphology compared with control eggs, and the eggs were intact and full (Figs. 3, 4). *Annona* species extract also affected the eggs of *Haemonchus contortus*; the eggs treated with the extract contained more liquid than control eggs and egg embryo cells shrank in contrast to full egg embryo cells in controls^20^. In order to ensure the accuracy of the results, we counted the rupture of egg shells and the number of larvae hatching after 100 *F. occidentalis* eggs were treated with spirotetramat. The results showed that the 0-h-old eggs did not hatch normally (Figs. 5, 6). It is inferred from this that spirotetramat may act on the shell of *F. occidentalis* eggs, destroying egg shell structure and causing it to rupture, thereby preventing normal hatching.

The *F. occidentalis* eggs treated with spirotetramat showed color darkening and separation between the egg body and the egg shell after 24 h of embryo development, and the egg shell broke after 36 h (Fig. 8). In further study of the effect of spirotetramat on *F. occidentalis* eggs, SEM imaging showed that the egg surface was shrunken, the pores were sunken and the outer chorion fell off. The eggs of *Aedes aegypti* were seriously deformed and degenerated after being treated with ovicidal lectins from *Moringa oleifera* and *Myracrodruon urundeuva*: the outer chorion of the egg shell disappeared, the nodules were few and deformed and the egg surface was covered with dense aggregates^21^. Cotton leafworm eggs treated with mahlab (*Prunus mahaleb* L.) kernel oil showed abnormal granular protrusions or protrusions on the egg surface, and even rupture of the chorionic surface; the egg surface wrinkles increased after mahlab oil treatment^22^. We speculate that spirotetramat affected the egg shells of *F. occidentalis*. The surface shrinkage and outer chorion loss may be due to egg shell damage by spirotetramat, which allows spirotetramat to enter the egg, and the sinking of the egg hole may be because the spirotetramat then affects embryonic development. Further study on the internal structure of *F. occidentalis* eggs showed that eggs treated with spirotetramat were flocculent, the internal protoplasm and yolk structures of the eggs were unclear, and flocculation in the protoplasm appeared to aggregate and form blocks (Fig. 12b). The egg shell structure was indistinct, and clear outer egg shell, inner egg shell, yolk membrane and lamella structures were not observed. The egg shell was also filled with a large amount of floccule (Fig. 12d).

These results indicate that spirotetramat has ovicidal activity on *F. occidentalis* and should have the same effect in actual production. Spirotetramat broke through the egg shell barrier of *F. occidentalis*, causing the egg shell to crack and deform. After spirotetramat entered the egg, the structure of the egg was destroyed, which affected embryo development and caused the larva to die before hatching. Therefore, spirotetramat can achieve a control effect before the larvae feed on plants, which is very important information for farmers. In actual production, eggs can be used as the application target for pest control. On the one hand, this circumvents the characteristics of fast, easy and concealed action of *F. occidentalis* nymphs and adults. On the other hand, it can prevent and control *F. occidentalis* before feeding.

## Data Availability

Data transparency.
